# Atrial high rate episodes in patients with cardiac implantable electronic devices: implications for clinical outcomes

**DOI:** 10.1007/s00392-019-01432-y

**Published:** 2019-02-13

**Authors:** Kazuo Miyazawa, Daniele Pastori, Yan-Guang Li, Orsolya Székely, Farhan Shahid, Giuseppe Boriani, Gregory Y. H. Lip

**Affiliations:** 1 Institute of Cardiovascular Sciences, University of Birmingham, Birmingham, UK; 2 Department of Internal Medicine and Medical Specialties, I Clinica Medica, Atherothrombosis Center, Sapienza University of Rome, Rome, Italy; 3 Department of Cardiology, Chinese PLA Medical School, Chinese PLA General Hospital, Beijing, China; 4 Cardiology Division, Department of Biomedical, Metabolic and Neural Sciences, University of Modena and Reggio Emilia, Policlinico di Modena, Modena, Italy; 5 Liverpool Centre for Cardiovascular Science, University of Liverpool and Liverpool Heart and Chest Hospital, Liverpool, UK; 6 Thrombosis Research Unit, Department of Clinical Medicine, Aalborg University, Aalborg, Denmark

**Keywords:** Atrial fibrillation, Atrial high rate episode, Cardiac implanted electronic device, Risk stratification scheme

## Abstract

**Background:**

Atrial high rate episodes (AHREs) detected by cardiac implantable electronic devices (CIEDs) are associated with an increased risk of stroke. However, the impact of AHRE on improving stroke risk stratification scheme remains uncertain.

**Objective:**

The purpose of this study was to assess the impact of AHRE on prognosis in relation with cardiovascular events and risk stratification.

**Methods:**

A total of 856 consecutive patients who had dual-chamber CIEDs implanted were retrospectively analyzed. To detect AHREs, they were monitored for 6 months after CIEDs’ implantation and were followed for a mean of 4.0 years for clinical outcomes such as thromboembolism or death.

**Results:**

Overall, 125 (14.6%) of patients developed AHREs within the first 6 months (median age 72.0 years, 39.3% female). Patients with AHREs had a high rate of thromboembolism (2.6%/year) and mortality (3.0%/year). On multivariate analysis, AHRE was significantly associated with increased risk of thromboembolism [hazard ratio (HR) 3.40; 95% confidence interval (CI) 1.38–8.37, *P* = 0.01] and death (HR 3.47; 95% CI 1.51–7.95; *P* < 0.01). The predictive abilities of the CHADS_2_ and CHA_2_DS_2_-VASc scores were modest, with no significant improvements by adding AHRE to those scores. However, the integrated discrimination improvement and net reclassification improvement showed that the addition of AHRE to the CHADS_2_ and CHA_2_DS_2_-VASc scores statistically improved their predictive ability for the composite outcome.

**Conclusions:**

AHRE was an independent factor associated with increased risk of clinical outcomes. The addition of AHRE to the clinical risk scores significantly improved discrimination for thromboembolism or death.

**Electronic supplementary material:**

The online version of this article (10.1007/s00392-019-01432-y) contains supplementary material, which is available to authorized users.

## Introduction

Atrial fibrillation (AF) is the most common sustained arrhythmia, and is associated with increased risks of morbidity and mortality [1]. Integrated management including anticoagulation therapy in patients with AF is increasingly recognized [2, 3], but there are still a substantial number of AF patients admitted with stroke, heart failure, and other complications [4, 5]. This can be to some extent due to asymptomatic nature of AF as approximately one-third of patients did not report any symptoms commonly attributable to AF (e.g., palpitations, shortness of breath, or chest pain) [6], leading to a prolonged delay in AF diagnosis and timely initiation of anticoagulation therapy. In many cases, asymptomatic AF is diagnosed only after the onset of complications such as ischemic stroke or congestive heart failure has occurred [7].

Subclinical and asymptomatic atrial tachyarrhythmias often precede the development of clinical AF, and can be detected by continuous cardiac monitoring technology including cardiac implantable electronic devices (CIEDs, e.g., permanent pacemakers, implantable cardioverter defibrillators [ICD], and cardiac resynchronization therapy [CRT] devices) [8]. Previous studies demonstrated that atrial high rate episodes (AHREs) detected by CIEDs have a high correlation with clinically documented AF [9], and are independently associated with an increased risk of ischemic stroke and systemic embolism [10–12].

Various efforts have been made to identify the risk factors for the development of AHRE and to assess the relationship between AHREs and clinical outcomes in patients with CIEDs. Although recent studies have investigated the impact of AHRE on the management of patient with AHREs, the optimal management of such patients remains uncertain in the current clinical guidelines, especially anticoagulation therapy in patients with AHREs [13].

In this study, we aimed to investigate the clinical characteristics of AHRE in a ‘real-world’ cohort of patients with CIEDs, and assessed the impact of AHRE on prognosis in relation with cardiovascular events and clinical event-risk stratification schemes.

## Methods

### Study population

The consecutive patients receiving pacemaker, ICD, and CRT devices, who attended the cardiology department of Sandwell and West Birmingham Hospitals NHS Trust (Sandwell General Hospital and City Hospital) in Birmingham, United Kingdom were retrospectively enrolled. In the present study, patients with single-chamber CIEDs (i.e., VVI and AAI devices) and patients with < 6 months of follow-up were excluded.

We retrospectively reviewed the patients’ medical records, and collected clinical information on demographics, co-morbidities, and concomitant medications. Prior history of AF was defined as a documented AF on 12-lead ECG or Holter ECG monitoring.

### Atrial high rate episode and clinical outcomes

The device diagnostic information was interrogated to assess whether patients had developed AHREs or not within the first 6 months since the time of CIEDs’ implantation. All CIEDs were programmed to the nominal setting, which detected any episodes of arrhythmia. We defined the AHRE as an episode lasting at least 5 min with atrial rate ≥ 175 beats per minute, given that previously published studies suggested that the 5 min cut-off value excluded most episodes of over-sensing due to mechanical problems and appropriately detected clinical AF [9]. Device diagnostic information on AHREs was reviewed by at least 1 experienced electrophysiologist, blinded to clinical outcomes.

The endpoint for the present study was the occurrence of thromboembolism (ischemic stroke, transient ischemic attack [TIA], or systemic embolism) or all-cause death 6 months after CIEDs’ implantation. Baseline characteristics of patients with and without AHREs were compared, and the predictive ability of CHADS_2_ and CHA_2_DS_2_-VASc scores for clinical outcomes was assessed. This study was conducted in accordance with the EU Guidance on Good Clinical Practice CPMP/ ECH/135/95. The present study was approved by the local research ethics committee and complied with the Declaration of Helsinki. All patients included in the study had there data anonymised.

### Statistical analysis

Continuous variables were presented as means and standard deviations (SD), unless not normally distributed, in which case medians and interquartile ranges (IQR) were used. Categorical variables were presented as frequencies and percentages. Censoring was done for the first event recorded. We compared categorical variables using Chi-square test and continuous variables using independent samples *t* test for normally distributed data or Mann–Whitney *U* test for non-normal distribution. Baseline characteristics, stroke risk profiles, and medications were tabulated between patients with and without AHREs. Annual incidence rates for the composite and individual endpoints were recorded in patients with and without AHREs. Kaplan–Meier survival curves were depicted, and differences were assessed by log-rank test. The independent effects of AHRE on the clinical outcomes were assessed using a Cox proportional hazards regression model including components of the CHA_2_DS_2_-VASc score (age assessed as a continuous variable), prior history of AF, and oral anticoagulant (OAC) use as co-varieties.

Receiver-operating characteristic (ROC) curve analysis was performed to test the predictive discrimination of the risk scores for clinical outcomes based on an area under the ROC curve (AUC). To compare the predictive ability of the predictive models, we calculated the statistical difference between the AUCs with the method of DeLong et al. [14] Furthermore, improvements in the predictive accuracy of the models were evaluated by calculating the integrated discrimination improvement (IDI) and the net reclassification improvement (NRI), as described by Pencina et al. [15].

We also assessed the clinical usefulness and net benefit of the predictive models using decision curve analysis (DCA), as described by Vickers et al. [16]. This analysis identifies patients who will have any of the adverse events evaluated, based on the predictions of the modified risk score in comparison with the original. The clinical net benefit is calculated by summing the benefits (true positive) and subtracting the harms (false positive). The result of this analysis is presented with the selected probability threshold plotted on the *x*-axis and the benefit of the evaluated model on the *y*-axis.

All statistical analyses were performed using IBM SPSS Statistics version 24.0 software (IBM Corp) and R software packages version 3.5.1 (R Development Core Team). Statistical significance was set as a two-sided *P* value of < 0.05.

## Results

### Baseline characteristics

Baseline characteristics of patients with and without AHREs are shown in Table 1. Median age of the patients was 72.0 (IQR: 62.0–80.0) years, and 336 (39.3%) were female. Of 856 patients with CIEDs, 74.6% had pacemaker, 15.0% ICD, and 10.4% CRT. During a mean follow-up of 48.2 ± 32.3 months, 125 (14.6%) of patients developed AHREs in the first 6 months. Patients with AHREs were older, with a higher prevalence of prior AF (and accordingly higher use of oral anticoagulants and digoxin, lower use of antiplatelets), compared to those without AHREs. No significant differences in mean CHADS_2_ and CHA_2_DS_2_-VASc scores were found between two groups.

**Table 1:** Baseline characteristics of patients with and without AHRE

	Overall (*n* = 856)	AHRE (*n* = 125)	No AHRE (*n* = 731)	*P* value
Demographics				
Age, median (IQR)	72.0 (62.0–80.0)	74 (63.0–81.0)	71.0 (62.0–79.0)	0.03
Age 65–74 y	232 (27.1)	29 (23.2)	203 (27.8)	0.29
Age > 75 y	383 (44.7)	66 (52.8)	317 (43.4)	0.05
Female gender [no., (%)]	336 (39.3)	342 (33.6)	294 (40.2)	0.16
BMI, median (IQR)	27.9 (24.6–31.7)	27.4 (23.5–31.8)	28.0 (24.6–31.5)	0.41
Medical history [no., (%)]				
Hypertension	603 (70.4)	91 (72.8)	512 (70.0)	0.53
Diabetes mellitus	241 (28.2)	36 (28.8)	205 (28.0)	0.86
Dyslipidemia	554 (68.6)	81 (68.6)	473 (68.7)	1.00
Heart failure	214 (25.0)	39 (31.2)	175 (23.9)	0.08
Prior stroke/TIA	92 (10.7)	15 (12.0)	77 (10.5)	0.63
Vascular disease	317 (37.0)	48 (38.4)	269 (36.8)	0.73
Prior history of AF	212 (24.8)	75 (60.0)	137 (18.7)	< 0.001
Thromboembolic risk, mean ± SD				
CHADS_2_ score	1.9 ± 1.2	2.0 ± 1.3	1.9 ± 1.2	0.07
CHA_2_DS_2_-VASc score	3.4 ± 1.6	3.5 ± 1.7	3.3 ± 1.6	0.22
Medications [no., (%)]				
Beta-blocker	270 (35.5)	47 (40.9)	223 (34.5)	0.19
ACE-I/ARB	436 (56.7)	66 (57.4)	370 (56.6)	0.87
Diuretics	276 (35.8)	42 (36.5)	234 (35.7)	0.87
Statin	509 (66.0)	74 (64.3)	435 (66.3)	0.68
OAC	151 (19.7)	54 (47.0)	97 (14.9)	< 0.001
Antiplatelet	396 (51.4)	49 (42.2)	347 (53.0)	0.03
Digoxin	38 (4.9)	12 (10.4)	26 (4.0)	0.003

*ACE-I* angiotensin-converting enzyme inhibitor, *AF* atrial fibrillation, *AHRE* atrial high rate episode, *ARB* angiotensin II receptor blocker, *BMI* body mass index, *IQR* interquartile range, *OAC* oral anticoagulant, *TIA* transient ischemic attack

### Clinical outcomes and atrial high rate episode

During the follow-up, the observed rates of thromboembolism, all-cause death and composite outcome were 4.2% (*n* = 36), 5.4% (*n* = 46) and 9.3% (*n* = 80), respectively (Table 2). Patients with AHREs had a higher risk of thromboembolism (incidence 2.6%/year) compared with those without AHREs (incidence 0.9%/year) [hazard ratio (HR) 2.703, 95% confidence interval (CI) 1.52–4.81, *P* < 0.001]. Similarly, patients with AHREs had higher risk of all-cause death and composite outcome (thromboembolism or all-cause death) than those without AHREs (3.0%/year vs. 1.1%/year, HR 3.85, 95% CI 1.90–7.80, *P* < 0.001 for all-cause death; 5.4%/year vs. 2.0%/year, HR 3.32, 95% CI 1.95–5.65, *P* < 0.001 for the composite outcome). In a subgroup analysis of patients who had no prior history of AF, patients with AHREs tended to have a higher risk of thromboembolism compared with those without AHREs, but not statistically significant. On the other hand, patients with AHREs had a significantly higher risk of all-cause death compared with those without AHREs (Supplementary Table 1).

**Table 2:** Clinical outcomes after first 6 months in patients with and without AHRE

Clinical outcomes	Overall (*n* = 856) [no. (%)]	Patients with AHRE (*n* = 125)		Patients without AHRE (*n* = 7310		Unadjusted HR (95% CI)	*P* value
		No. of events	%/year	No. of events	%/year		
Thromboembolism	36 (4.2)	9	2.6	27	0.9	2.703 (1.52–4.81)	< 0.001
All-cause death	46 (5.4)	11	3.0	35	1.1	3.845 (1.90–7.80)	< 0.001

*AHRE* atrial high rate episode, *CI* confidence intervals, *HR* hazard ratio

**Fig. 1:**
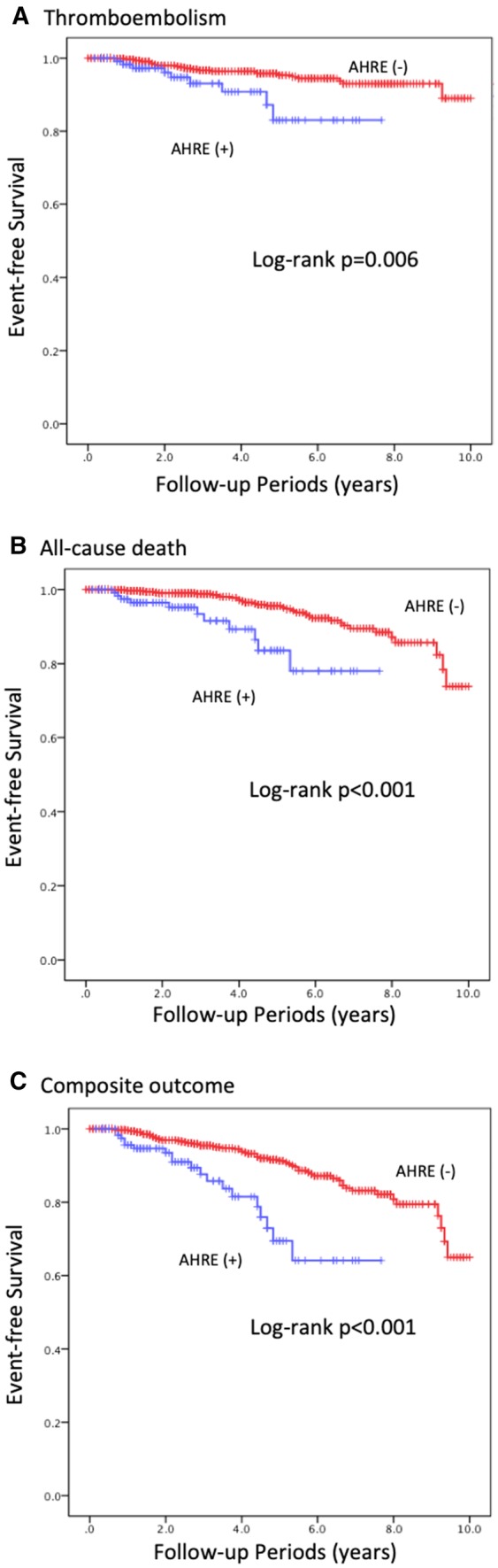
Kaplan–Meier curve analysis for thromboembolism (**a**), all-cause death (**b**), and composite outcome (**c**)

Kaplan–Meier curve analysis shows that crude event-free survival for thromboembolism, all-cause death and composite outcome appeared to be lower in patients with AHREs than those without AHREs (*P* = 0.006, *P* < 0.001, *P* < 0.001, respectively) (Fig. 1). On multivariate Cox proportional hazards regression analysis (Table 3), age was significantly associated with increased risk of all-cause death and the composite outcome (HR 1.13, 95% CI 1.08–1.18, *P* < 0.001, HR 1.06, 95% CI 1.03–1.09, *P* < 0.001, respectively); heart failure with all-cause death (HR 2.21, 95% CI 1.03–4.74, *P* = 0.04); prior stroke or TIA with thromboembolism and composite outcome (HR 2.64, 95% CI 1.16–5.99, *P* = 0.02, HR 2.23, 95% CI 1.25–3.96, *P* = 0.006, respectively). Prior history of AF was not an independent factor associated with any outcomes (all *P* > 0.05). Similarly, the use of OAC was not significantly associated with any outcomes (all *P* > 0.05), although the association trended towards being protective.

**Table 3:** Multivariable Cox regression analysis for clinical outcomes

Outcomes and variables	Thromboembolism		All-cause death		Composite outcome	
	HR (95% CI)	*P* value	HR (95% CI)	*P* value	HR (95% CI)	*P* value
Age	1.01 (0.98–1.04)	0.54	1.13 (1.08–1.18)	< 0.001	1.06 (1.03–1.09)	< 0.001
Female gender	1.01 (0.48–2.13)	0.97	1.16 (0.60–2.25)	0.65	1.17 (0.71–1.93)	0.54
Hypertension	1.28 (0.50–3.26)	0.61	0.80 (0.36–1.79)	0.59	0.97 (0.53–1.77)	0.91
Diabetes mellitus	1.50 (0.73–3.08)	0.27	1.68 (0.86–3.28)	0.13	1.56 (0.96–2.54)	0.07
Heart failure	0.60 (0.24–1.53)	0.29	2.20 (1.03–4.74)	0.04	1.31 (0.73–2.35)	0.37
Prior stroke/TIA	2.64 (1.16–5.99)	0.02	2.17 (0.99–4.73)	0.05	2.23 (1.25–3.96)	0.01
Vascular disease	1.64 (0.78–3.42)	0.19	1.03 (0.50–2.10)	0.94	1.28 (0.77–2.14)	0.34
Prior history of AF	1.08 (0.43–2.71)	0.88	0.95 (0.42–2.18)	0.91	0.97 (0.52–1.80)	0.92
OAC use	0.66 (0.24–1.81)	0.42	0.50 (0.19–1.32)	0.16	0.51 (0.25–1.03)	0.06
AHRE	3.40 (1.38–8.37)	0.01	3.47 (1.51–7.95)	0.003	3.52 (1.89–6.55)	< 0.001

Adjusted covariates including components of the CHA_2_DS_2_-VASc score (age assessed as a continuous variable), prior AF documentation, OAC use, and AHRE lasting at least 5 min

*AF* atrial fibrillation, *AHRE* atrial high rate episode, *CI* confidence intervals, *HR* hazard ratio, *OAC* oral anticoagulant, *TIA* transient ischemic attack

On multivariate adjustment, AHRE was significantly associated with increased risk of all outcomes (HR 3.40, 95% CI 1.38–8.37, *P* = 0.008 for thromboembolism, HR 3.47, 95% CI 1.51–7.95, *P* = 0.003 for all-cause death, and HR 3.52, 95% CI 1.89–6.55, *P* < 0.001 for the composite outcome).

When we performed a subgroup analysis of patients without prior history of AF (*n* = 644) and those with prior history of AF (*n* = 212), multivariate Cox regression analysis showed that the impact of AHRE slightly weakened rather than entire population, but still significant on the clinical outcomes, especially all-cause death and the composite outcome (Supplementary Tables 2 and 3).

### Adding AHRE to the CHADS_2_ and CHA_2_DS_2_-VASc scores for predicting clinical outcomes

ROC curve analysis showed that the predictive abilities of the CHADS_2_ and CHA_2_DS_2_-VASc scores were modest and there were no significant improvements after adding AHRE to the CHADS_2_ and CHA_2_DS_2_-VASc scores (CHADS_2_: AUCs 0.60 to 0.61, *P* = 0.29 for thromboembolism; AUCs 0.65 to 0.65, *P* = 0.73 for all-cause death; and AUCs 0.63 to 0.64, *P* = 0.32 for the composite outcome, CHA_2_DS_2_-VASc: AUCs 0.60 to 0.61, *P* = 0.35 for thromboembolism; AUCs 0.68 to 0.68, *P* = 0.68 for all-cause death; and AUCs 0.65 to 0.65, *P* = 0.33 for the composite outcome) (Table 4).

**Table 4:** Comparison of the ROC curve, IDI and NRI of the CHADS_2_ vs. CHADS_2_ + AHRE and CHA_2_DS_2_-VASc vs. CHA_2_DS_2_-VASc + AHRE in predicting outcomes

Clinical outcomes and risk scores	C-statistic	95% CI	*P* value*	IDI	*P* value	NRI	*P* value
Thromboembolism							
CHADS_2_	0.56	0.50–0.69	0.29	0.002	0.09	0.23	0.12
CHADS_2_ + AHRE	0.61	0.52–0.70					
CHA_2_DS_2_-VASc	0.60	0.52–0.68	0.35	0.002	0.08	0.22	0.18
CHA_2_DS_2_-VASc + AHRE	0.61	0.53–0.69					
All-cause death							
CHADS_2_	0.65	0.58–0.72	0.73	0.004	0.100	0.20	0.13
CHADS_2_ + AHRE	0.65	0.58–0.72					
CHA_2_DS_2_-VASc	0.68	0.60–0.75	0.68	0.004	0.11	0.20	0.13
CHA_2_DS_2_-VASc + AHRE	0.68	0.61–0.75					
Composite outcome							
CHADS_2_	0.63	0.57–0.69	0.32	0.01	0.03	0.20	0.04
CHADS_2_ + AHRE	0.64	0.58–0.70					
CHA_2_DS_2_-VASc	0.65	0.59–0.70	0.33	0.01	0.02	0.20	0.04
CHA_2_DS_2_-VASc + AHRE	0.65	0.60–0.71					

*AHRE* atrial high rate episode, *CI* confidence interval, *IDI* integrated discriminatory improvement, *NRI* net reclassification improvement, *ROC* receiver-operating characteristic

*For C-statistic comparison

Based on the IDI and the NRI, the addition of AHRE to the CHADS_2_ and CHA_2_DS_2_-VASc scores statistically improved discriminative value for composite outcome (CHADS_2_: IDI 0.01, *P* = 0.03; NRI 0.20, *P* = 0.04, CHA_2_DS_2_-VASc: IDI 0.01, *P* = 0.02; NRI 0.20, *P* = 0.04), but not for thromboembolism and all-cause death (CHADS_2_: IDI 0.002, *P* = 0.09; NRI 0.23, *P* = 0.12, for thromboembolism, IDI 0.004, *P* = 0.10; NRI 0.20, *P* = 0.13, for all-cause death, CHA_2_DS2-VASc: IDI 0.002, *P* = 0.08; NRI 0.22, *P* = 0.18, for thromboembolism; IDI 0.004, *P* = 0.11; NRI 0.20, *P* = 0.13, for all-cause mortality) (Table 4).

**Fig. 2:**
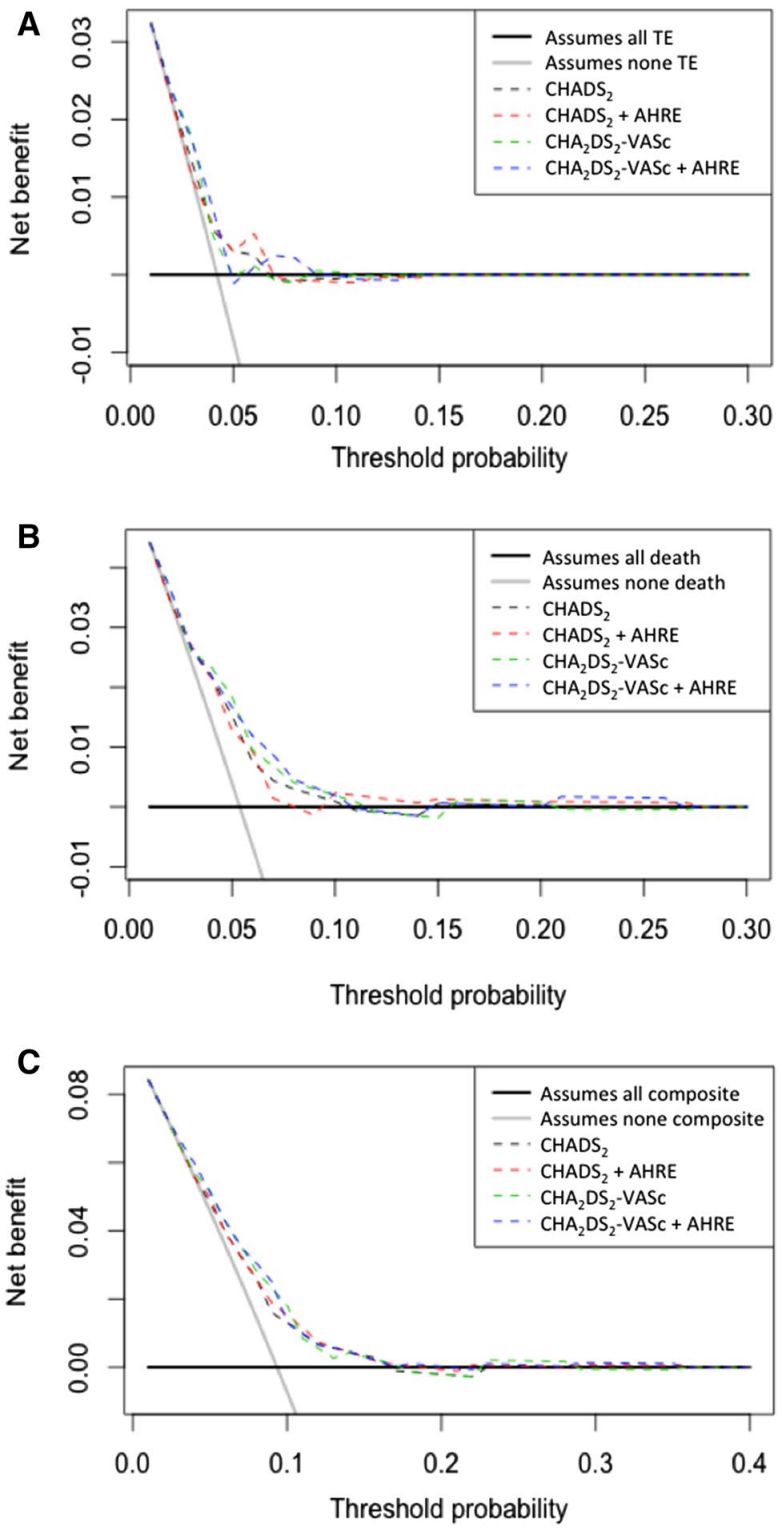
Decision curve analysis for predicting cardiovascular events (**a** thromboembolism, **b** all-cause death, and **c** composite outcome)

DCA graphically demonstrated that there were minimal net benefits of the addition of AHRE to the CHADS_2_ and CHA_2_DS_2_-VASc scores for predicting thromboembolism (Fig. 2a), all-cause death (Fig. 2b), and composite outcome (Fig. 2c).

## Discussion

The main finding of the present study is that AHRE detected by CIEDs was an independent factor associated with significantly increased risks of thromboembolism and all-cause death, regardless of the presence or absence of prior history of AF. Second, the addition of AHREs to the CHA_2_DS_2_-VASc score statistically improved its discrimination ability for the composite outcome of thromboembolism or all-cause death. This is a contemporary ‘real-world’ cohort of patients with CIEDs, in relation with AHRE. To the best of our knowledge, no previous study has investigated the clinical impact of AHRE on risk stratification for thromboembolism and all-cause death.

The widespread use of the CIED technology in the management of a broad spectrum of cardiac diseases (i.e., bradycardia, life-threatening tachycardia, and heart failure) offers the long-term continuous ECG monitoring, which allows an early detection of atrial tachyarrhytmias including AF before they become clinically evident [17]. However, most of AHREs are asymptomatic, short-lasting, and hard to be detected by the conventional methods such as 12-lead ECG or ambulatory Holter ECG monitoring. The reported incidence of AHRE in patients with CIEDs is relatively variable across studies, ranging from 30 to 70% [18]. Although the incidence may strongly depend on the clinical profile of study population, the previous studies have consistently reported that AHRE is associated with a substantial risk of subsequent development of clinically diagnosed AF, and is also associated with an increased risk of ischemic stroke and death [10–12]. However, the absolute risk of stroke in patients with AHREs may be lower than in those with clinical AF.

In general, AF is known to be associated with fivefold risk of stroke compared to normal sinus rhythm, while recent meta-analysis showed that the annual rate of stroke in patients with AHREs was 1.89/100 person-year with 2.4-fold increased risk of stroke compared to those without AHREs [19]. In the present study, AHRE was significantly associated with a threefold greater risk of thromboembolism and of all-cause death. Our findings reinforce the evidence that AHRE may not hold the same adverse prognosis as clinical AF, but may be considered as an early stage of clinical AF carrying an intermediate risk of stroke. On the other hand, the incidences of clinical outcomes in the present study were relatively higher than the previous studies including general AF population. This may be due to the differences in baseline medical history, as in our study, half of patients were elderly (i.e., > 75 years) with a high proportion of hypertension, dyslipidemia, and vascular disease. The present study included patients with not only pacemaker, but also those with ICD and CRT, who frequently have left ventricular dysfunction and/or ischemic heart disease and are at risk of sudden cardiac death.

Interestingly, we found that prior history of clinical AF was more frequently observed in patients with AHREs, but this was not independent factor associated with any outcomes in Cox proportional hazards regression models. This can be explained by the fact that CIEDs’ implantation itself could suppress new AF development. Previous studies have demonstrated that dual-chamber pacing modes reduce the incidence of AF in patients with sick sinus syndrome [20, 21]. In patients with CRT, hemodynamic improvement due to synchronized bi-ventricular pacing is reported to decrease the incidence of AF [22]. Furthermore, in a previous study of a heart failure cohort, there was no significant difference in the cumulative probability of developing thromboembolic events between AHRE patients with and without history of AF [23]. Of note, the subgroup analysis of patients without prior history of AF demonstrated that we found a significant association between AHRE and clinical outcomes, although there were a relatively small number of patients and clinical events. Thus, current actual AF burden (i.e., AHRE) has a more significant impact on clinical outcomes rather than a previously documented history of AF.

In the present study, we showed the modest predictive abilities of the CHADS_2_ and the CHA_2_D_2_-VASc scores for risk of clinical outcomes, even in the subgroup of patients with and without prior history of AF. Possible explanations for the modest predictive performance in the CIED population include the differences in baseline medical history between the general AF population and CIEDs’ population, and the suppression of new AF episodes after CIEDs’ implantation, which may affect the predictive ability of clinical risk scores in this patient population. However, AHRE has previously been reported to help to refine event-risk stratification in such patients. Botto et al. demonstrated that combination of the data on AHRE with the CHADS_2_ score divided patients with CIEDs into two subpopulations with significantly different risks of thromboembolic events [24]. Furthermore, Boriani et al. reported that the addition of AHRE burden to the CHADS_2_ and CHA_2_DS_2_-VASc scores significantly improved their C-statistics [25]. In the present study, ROC curve analysis indicated no significant improvements of C-statistics by adding the data on AHREs to the clinical risk scores, but we showed a statistically significant improvement of its discriminatory value and a net clinical benefit using the IDI, NRI and (minimally) DCA. The present study can provide novel insights into the current management in this field, among ‘real-world’ patients with CIEDs.

Current guidelines do not address in detail the management of patients with AHREs. Therefore, further studies are needed to explore the role of AHRE for event-risk stratification and decision-making for thromboprophylaxis in AHRE. Some prospective clinical trials are ongoing, which are investigating the benefit of OACs in patients with CIEDs, and will provide useful information on management of patients with AHREs [26, 27].

### Limitations

There are several limitations in the present study. First, this is a single-center, retrospective, and observational study in a hospital-based setting. A relatively small number of patients were included in the present study, which may have reduced the detection power and possibly influenced the validity of some interactions. Second, we collected the clinical data at the time of CIEDs’ implantation, while the data at follow-up were not taken into account. As expected, initiation and discontinuation of OAC during follow-up would affect clinical outcomes, and we had limited information on this. Finally, data on quality of anticoagulation such as international normalized ratio or time in therapeutic range were not available in the present study. Notwithstanding the relatively modest size and residual confounding, we found no significant relationship between OAC use and thromboembolism in multivariate Cox regression analysis.

## Conclusion

In conclusion, AHRE was an independent factor associated with increased risk of clinical outcomes. The addition of AHRE to the CHADS_2_ and CHA_2_DS_2_-VASc scores significantly improved discrimination for thromboembolism or death.

## Electronic supplementary material

Below is the link to the electronic supplementary material.

Supplementary material 1 (DOCX 75 KB)
