# Supplementary material for: Atrial high rate episodes in patients with cardiac implantable electronic devices: implications for clinical outcomes
Source: Clin Res Cardiol. 2019 Feb 13;108(9):1034–41. doi: 10.1007/s00392-019-01432-y (PMC6694071; doi:10.1007/s00392-019-01432-y)
Supplement: Supplementary file 1 — Supplementary material 1 (DOCX 75 KB) [file 392_2019_1432_MOESM1_ESM.docx]

Supplementary table 1. Clinical outcomes after first 6 months in subgroup of patients with and without prior history of AF

| Clinical outcomes | Overall  (No. [%]) | Patients with AHRE  n=50 | | Patients without AHRE  n=594 | | Unadjusted HR (95% CI) | p value |
| --- | --- | --- | --- | --- | --- | --- | --- |
|  |  | No. of events | %/year | No. of events | %/year |  |  |
| Patients without prior history of AF (n=644) | | | | | | | |
| Thromboembolism | 26 (4.0) | 3 | 2.3 | 23 | 0.9 | 2.655 (0.79-8.96) | 0.115 |
| All-cause death | 33 (5.1) | 4 | 2.9 | 29 | 1.1 | 3.857 (1.32-11.28) | 0.014 |
| Patients with prior history of AF (n=212) | | | | | | | |
| Thromboembolism | 10 (4.7) | 6 |  | 4 |  | 3.121 (0.88-11.10) | 0.079 |
| All-cause death | 13 (6.1) | 7 |  | 6 |  | 3.450 (1.08-11.01) | 0.037 |

AHRE; atrial high rate episode, CI; confidence intervals, HR; hazard ratio

Supplementary table 2. Multivariable Cox regression analysis for clinical outcomes in patients without prior history of AF*

| Outcomes and variables | Thromboembolism | | All-cause death | | Composite outcome | |
| --- | --- | --- | --- | --- | --- | --- |
|  | HR (95% CI) | p value | HR (95% CI) | p value | HR (95% CI) | p value |
| Age | 1.02 (0.98-1.05) | 0.40 | 1.13 (1.07-1.18) | <0.001 | 1.06 (1.03-1.10) | <0.001 |
| Female gender | 0.81 (0.33-2.00) | 0.64 | 1.26 (0.57-2.77) | 0.57 | 1.15 (0.64-2.07) | 0.64 |
| Hypertension | 1.11 (0.39-3.19) | 0.84 | 1.54 (0.44-5.33) | 0.50 | 1.25 (0.57-2.73) | 0.59 |
| Diabetes mellitus | 1.30 (0.55-3.08) | 0.55 | 1.89 (0.87-4.12) | 0.11 | 1.49 (0.84-2.62) | 0.17 |
| Heart failure | 0.85 (0.26-2.78) | 0.79 | 2.47 (0.91-6.70) | 0.08 | 1.68 (0.80-3.55) | 0.17 |
| Prior stroke/TIA | 3.39 (1.30-8.82) | 0.01 | 2.23 (0.82-6.07) | 0.12 | 2.76 (1.39-5.48) | 0.004 |
| Vascular disease | 1.25 (0.49-3.19) | 0.63 | 0.96 (0.40-2.29) | 0.93 | 1.09 (0.58-2.05) | 0.79 |
| OAC use | 0 (0) | 0.98 | 0.46 (0.10-2.17) | 0.33 | 0.26 (0.06-1.11) | 0.07 |
| AHRE | 3.48 (0.99-12.16) | 0.05 | 3.46 (1.13-10.60) | 0.03 | 3.62 (1.56-8.37) | 0.003 |

*Adjusted covariates including components of the CHA_2_DS_2_-VASc score (age assessed as a continuous variable), OAC use and AHRE lasting at least 5min.

AF; atrial fibrillation, AHRE; atrial high rate episode, CI; confidence intervals, HR; hazard ratio, OAC; oral anticoagulant, TIA; transient ischemic attack

Supplementary table 3. Multivariable Cox regression analysis for clinical outcomes in patients with prior history of AF*

| Outcomes and variables | Thromboembolism | | All-cause death | | Composite outcome | |
| --- | --- | --- | --- | --- | --- | --- |
|  | HR (95% CI) | p value | HR (95% CI) | p value | HR (95% CI) | p value |
| Age | 0.99 (0.92-1.06) | 0.72 | 1.16 (1.04-1.29) | 0.01 | 1.05 (0.99-1.11) | 0.06 |
| Female gender | 1.70 (0.38-7.51) | 0.49 | 0.61 (0.14-2.62) | 0.51 | 0.94 (0.34-2.60) | 0.91 |
| Hypertension | 3.16 (0.35-28.75) | 0.31 | 0.21 (0.05-0.90) | 0.04 | 0.61 (0.21-1.72) | 0.35 |
| Diabetes mellitus | 1.58 (0.40-6.23) | 0.51 | 2.10 (0.42-10.40) | 0.36 | 2.05 (0.76-5.57) | 0.16 |
| Heart failure | 0.44 (0.08-2.42) | 0.35 | 1.28 (0.34-4.85) | 0.72 | 0.92 (0.34-2.52) | 0.92 |
| Prior stroke/TIA | 1.01 (0.20-4.96) | 0.99 | 2.69 (0.62-11.66) | 0.19 | 1.49 (0.51-4.29) | 1.49 |
| Vascular disease | 2.97 (0.77-11.47) | 0.11 | 1.07 (0.27-4.27) | 0.92 | 1.73 (0.68-4.43) | 0.25 |
| OAC use | 1.14 (0.30-4.36) | 0.85 | 0.32 (0.08-1.28) | 0.11 | 0.66 (0.26-1.65) | 0.37 |
| AHRE | 3.28 (0.81-13.34) | 0.10 | 3.11 (0.88-11.06) | 0.08 | 2.79 (1.10-7.06) | 0.03 |

*Adjusted covariates including components of the CHA_2_DS_2_-VASc score (age assessed as a continuous variable), OAC use and AHRE lasting at least 5min.

AF; atrial fibrillation, AHRE; atrial high rate episode, CI; confidence intervals, HR; hazard ratio, OAC; oral anticoagulant, TIA; transient ischemic attack
